# An examination of auxetic componentry for applications in human-centred biomedical product design settings

**DOI:** 10.1007/s12008-023-01682-1

**Published:** 2023-12-21

**Authors:** Lewis Urquhart, Francesco Tamburrino, Paolo Neri, Andrew Wodehouse, Craig Fingland, Armando Viviano Razionale

**Affiliations:** 1 University of Strathclyde, Glasgow, UK; 2 University of Pisa, Pisa, Italy; 3 Loud1Design, Glasgow, UK

**Keywords:** Auxetics, Mechanical testing, Biomedical applications, Human-centred design

## Abstract

This paper explores how the examination of additively manufactured auxetic componentry can be applied in human-centred design settings with particular focus on biomedical products. Firstly, the design applications of auxetics are detailed followed by a review of the key problems facing practical researchers in the field with the treatment of boundary conditions identified as a key issue. The testing setup that is then introduced utilises a novel method of part mounting and facilitates optical analysis and real-time force–displacement measurements. A study is advanced that analyses three different auxetic structures (re-entrant, chiral, and semi-rigid), a set of samples of which were additively manufactured in flexible TPU material. A range of parameters were varied across the three designs including interior geometry and wall thicknesses in order to demonstrate the effectiveness of the setup for the examination of the different structures. The results from these examinations are subsequently discussed and a number of suggestions made regarding how this kind of analysis may be integrated into novel design development workflows for achieving human-centred biomedical devices which often require detailed consideration of ergonomic and usability factors.

## Introduction and background

**Fig. 1:**
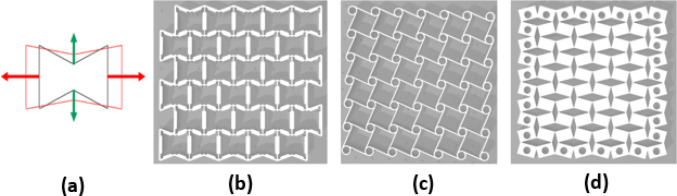
Force–deformation diagram for an auxetic structure (**a**), Typical re-entrant (**b**), chiral (**c**), and semi-rigid (**d**) auxetic geometry

Auxetics are presenting designers and engineers new possibilities in terms of product embodiments, manufacturing options and functions. With respect to biomedical applications, there is particular focus on how auxetics may offer localised functional features that could facilitate bespoke user-centred applications. Auxetic materials display a negative Poisson’s ratio when subjected to loads [1] and have been studied extensively over recent years within engineering science in a wide array of settings. There are many forms of auxetics and designs can occupy a micro or a macro space. Common designs include re-entrant, chiral, and semi-rigid [2] (Fig. 1) but there are also star and triangle variations [3] all of which can be embodied as larger physical objects or at the micro-scale within materials like auxetic foams [4]. This research will explore how information extracted from the testing and analysis of auxetics can feed into human-centred design contexts with particular focus on biomedical applications that usually require larger levels of focus around ergonomic and usability features.

Testing auxetics and other deformable structures with a view to advancing understanding of mechanical behaviours, exploring applications, or expanding the scope of possible testing strategies presents a distinct challenge for researchers. Notably, control and monitoring of buckling behaviours and boundary conditions has proved to be difficult to implement due to direct interference with the structures. For this reason, achieving setups that can replicate simulated results with reliability and accuracy is difficult. With this work, we seek to address some of these issues by examining the shortcomings of various practical testing approaches proposed throughout the literature and presenting results from a new testing setup and subsequently exploring the results. The new testing system developed by the authors explores a novel method for controlling the boundary conditions. Boundary conditions are a fundamental concern in this kind of mechanical testing as they affect how the overall structure responds to change such as large mechanical deformations from loading forces. The aim is to explore a novel testing approach based on a new setup for boundary and constraints conditions under bi-axial loading conditions. This paper will thus be split into three critical sections; Firstly we will explore the research literature regarding how auxetics and similar metamaterial structures are being utilised in new design settings with close attention paid to human-centred and biomedical contexts. Secondly, we will examine the different kinds of approaches other researchers have taken in the study of auxetic mechanical behaviour, thirdly we will present a set of results from a novel testing setup developed to address the shortcomings noted in the other setups described in the literature. Lastly, we will develop a discussion of these results with respect to human-centred and biomedical design applications with suggestions for how auxetics can fit into novel product development workflows.

### Design applications of auxetics

While auxetics clearly display interesting mechanical characteristics, the applications of these materials have proved a bit more limiting.

**Fig. 2:**
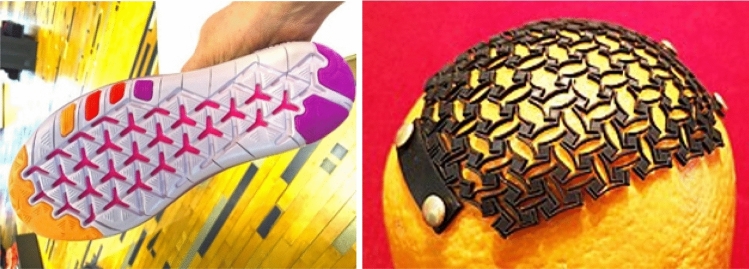
Examples of auxetics used in different product contexts (open-source images from Wikimedia Commons)

One prominent emerging area is footwear, especially sports footwear where areas of localised performance and functionality control are desirable. Figure 2 illustrates a typical example of how auxetics can be utilised in a shoe design setting whereby the auxetic pattern on the sole is configured to facilitate easier deformation when used, allowing the user better range of movement when running. While this is a generic example, some researchers have been exploring more advanced and technical configurations. In a review by Duncan and others [5], a range of applications for auxetics in sport settings are discussed. Of particular interest is the use of auxetic materials as a form of protective material such as padding or body armour. The review highlights how manufacturers of sportswear are consistently exploring new manufacturing methods as a means of creating more functional products more efficiently as the uptake of additive manufacturing technologies grows. For this reason, brands such as Under Armour have explored the use of re-entrant auxetic lattice structures in their footwear. Others such as Nike have patented auxetic foam technology that utilised rigid-rotating triangle auxetic lattices.

In terms of personal protective equipment, auxetics have been utilised to achieve greater fit around sensitive areas of the human body with complicated ergonomic requirements. Work from Mizzi and others for instance [6] developed a range of designs, one of which was shown to conform successfully to the spherical-type structures of the human skull, presenting possibilities for helmet designs (also see Fig. 2). Furthermore, Duncan and others [5] have highlighted how auxetics could be used in other protective contexts such as knee or abdomen padding (see work from Moroney for another example [7]) as well as the potential benefits of self-cleaning properties or dirt shedding due to multi-axis expansion features of NPR materials. This multi-axis expansion property may also be applied to clothing to increase breathability or be used to facilitate large changes in structure sizing which could benefit storage capability of product such as backpacks (see the work of Alderson et al. [8]).

Outside of sportswear, auxetics have been explored in other settings. Mercieca and others [9] for example have looked at the use of auxetics in footwear more generally and applied the structures to high-heeled shoes such as stilettoes to improve comfort for the wearer. Additional interest has been generated in applying auxetics within fashion and architecture. In a review by Papadopoulou and others [10], a range of interesting examples are cited in clothing design and structural building work that highlight how auxetic materials can conform to changes in the environment. Similarly, Konakovic and others [11] have described a variety of emerging design applications including conformal materials for clothing such as leatherwear with implicit auxetic patterning, Kagome lattice designs for complex structural mapping e.g., facial features and even household products like lighting in which a neutral and deformed auxetic state can conform to on–off “modes” for a lampshade. Other interesting work has explored how auxetics can be used for bio-inspired designs of products such as chairs and belts that have distinct aesthetic characteristics [12].

### Attuned ergonomic design: biomechanics and biomedical devices

While the work in auxetics is extensive and a full review is not the focus of this work, we do wish to consider how auxetics are being applied in biomedical settings. A range of work has considered the applications of auxetics in medical settings as according to an online lecture by Sheffield Hallam University [13] there are naturally occurring auxetic-type structures that exist already in the human body such as carotid arteries, Achilles tendon and stem cell nuclei, meaning that reapplying synthetically produced auxetics or metamaterials may yield extensive biomedical applications. In a review, Lvov and others [14] have described some of the experimental uses of auxetic materials in biomedical contexts. The morphing properties of auxetic materials are highlighted as of particular interest to researchers as this allows them to conform to complex, non-linear geometric conditions in addition to the attractive strength to weight ratios of many auxetic configurations.

Lvov and others [ibid] highlight several areas in which there has been extensive conceptual and practical work. Spinal surgery is noted as an emerging area of interest whereby "artificial intervertebral discs made of high-density auxetic polyethylene can bend and twist and may provide improved biomechanical performance compared with traditional disc replacement solutions” (p. 3). Additionally, an auxetic pedicle screw explored by Yan Yao et al. [15] is noted to have improved interaction mechanics between the bone and the screw structure. Similar concepts have been explored by Mardling [16] and Kim, Son and Lee [17] who have examined the use of auxetics for engineered tissue scaffolds in which cell proliferation and migration is enhanced and improved by and auxetic scaffold. Other notable areas of focus in auxetics application are medical arterial stents in which mechanical flexibility is a highly desirable design feature, hip implants, which use hybrid approaches of conventional and auxetic metamaterials, and cardiac patches in which re-entrant auxetics mechanics are utilised to mimic the motions of the heart muscles.

A large emerging area is that of orthotics, which requires careful articulation with the ergonomics of the user and a detailed understanding of the forces acting within the envelope of product interaction. In a notable study from Panico and others [18], a conceptual design for a neck-brace built from auxetic structures is explored in which the architecture of the brace conforms and flexes in line with the anatomical motions of the neck. The structure’s resistance to tensile and compressive forces made it ideal for this kind of application. A similar study has explored the design of an auxetic foam ankle brace that can promote healing in Achilles tendon injuries [19] and work from MIT has explored the use of flexible auxetic braces to support muscles and tendons [20].

### Conventional testing setups

Now that we have reviewed some of the applications of auxetics, we can now consider how the mechanics of auxetics are understood and tested. As we have seen, the context of use is important in understanding how the auxetic will respond mechanically, for this reason; the boundary condition of the test setup is of paramount importance. Within the literature focusing on the examination of 3D printed auxetic components, several setups have been identified that we could classify as “conventional”, requiring equipment that is less advanced and more freely accessible for the average researcher. Simple jigs for instance have been developed in order to examine the behaviour of parts under different loading conditions. The simple configuration seen in [21] encases the structure at its top and base making the mechanical loading application uniform, furthermore the transparency of the plastic allows for real-time visual examination of the deformation though no formal means of measuring the dimensional changes is advanced. Other researchers have explored the use of slightly more sophisticated setups with readily available componentry. There are notable examples in the literature of different methods of holding the parts within custom jigs. Research from [22] for instance uses a fixed jaw and a movable jaw in conjunction with an optical analysis setup. Markers are placed directly on the part to track the relative position of the points as the part is subjected to tension. The change in the relative position of the points is then interpreted by a computer, providing an assessment of how the part behaves mechanically. Moving the boundary condition away from the core structure being studied by using a set of nodes that can then be integrated into the jig, [23] explored an optical analysis method by tracking the relative position of numerous points on the overall auxetic structure. The setup used a jaw style jig that holds the component that is then pulled to tension using a series of rail-mounted stepper motors. Interestingly, a set of nodes have been added to the core structure that are then integrated into the jig, thus allowing the central structure to behave somewhat independently, moving the boundary problem away from the section being focused on directly. In another setup, the researchers [24] secured the sample with use of a set of supporting truss structures whereby a set of truss pins are placed uniformly within the auxetic structure itself. This means that the boundary condition can be more carefully controlled, and the setup potentially facilitates more complex local deformation tests although the boundary condition remains uncontrolled on two sides of the component. The researchers used a similar optical analysis method to determine the results.

### Advanced testing setups

Other researchers have utilised what we can categorise as “advanced” methods of analysis that go beyond the optical analysis methods considered in the previous section. For example, a low-speed hydraulic press was used in [25] to test auxetic samples. A uniform contact surface between the sample and the testing plates guaranteed controlled boundary conditions. The sample behaviour was assessed by measuring the compression speed rate of the material with an accelerometer. A less conventional approach was investigated in [2], where the auxetic specimen was tested off-axis. In this scenario, the 3D printed metallic specimen is subject to non-uniform solicitation. On the other hand, in [26] several different auxetic structures were tested from both a numerical and experimental point of view, which highlighted relevant difference in mechanical behaviour. Similar research investigated the reliability of different FE modelling software in this simulation field, demonstrating that strain behaviour is generally well described, while stress values are more critical to be accurately obtained [27]. The dynamics of AM lattice structure was studied in [28] through FE simulation. An extensive review analysis demonstrated that a lack of knowledge exists in the matching between the design process of such structures and the simulation strategy, leading to a poor design outcomes. A validation of optical experimental techniques with respect to numerical results was provide in [29, 30] during uniaxial testing. Finally, non-conventional auxetic structures were investigated in [31], concluding that “double-U” structures were found to be more robust with respect to manufacturing damage and stress concentrations when subject to elastic loading.

Taken together, we can see how there are a range of different testing strategies and associated issues to do with the control of the boundary conditions of test samples present problems for researchers. In the next sections, we will consider the development of a novel testing configuration that aims to address some of these boundary condition issues. Firstly, we will provide an overview of the new configuration design and then explore a set of results that were derived from this new setup.

## Development of a novel testing configuration

### Testing setup

**Fig. 3:**
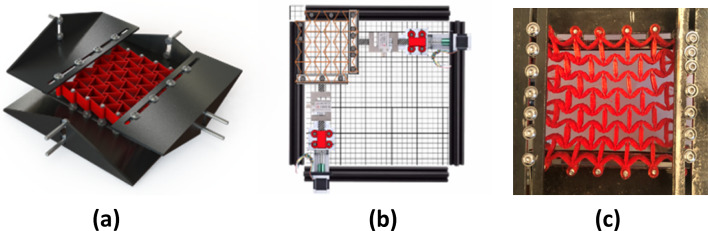
Design of fully integrated testing rig. Mounting design with support trusses and rails detail (**a**), schematic of jig with force gauge setup (**b**), Physical re-entrant sample mounted in testing jig (**c**)

With a view to exploring how the shortcomings of these testing setups could be addressed, we aimed to develop a novel setup. The main boundary condition problem has been addressed through the creation of a bespoke jig combined with the development of a specially designed testing rig and control interface see [32] for a full overview of the design development. Figure 3 below shows how the samples were mounted during testing. A series of adjustable truss pins were set into the sample on rails—this allowed both a small degree of movement but crucially held the sample firmly enough in order that the tension and compression forces could be applied. A basic oil-based lubricant was added to the rails to reduce the effects of friction. Taking inspiration from other research efforts (notably [24]) mounting points were set into the samples at uniformly distributed perimeter positions on all four sides of the auxetic sample. Recognizing that there are clear benefits to both the optical analysis and a mechanical analysis, the setup was configured to both record time lapsed images to record displacements and to measure the mechanical forces. As shown in Fig. 3a–c, sensitive force gauges were placed on two sides of the jig, recording real-time forces in the *X* and *Y* directions. The testing rig design was centred on mounting and deforming 70 × 70 mm auxetic samples as shown. Each test recorded the mechanical forces acting through the auxetic as the parts were slowly deformed 10 cm into a tensioned state and then reversed into a compression state by 10 cm.

**Fig. 4:**
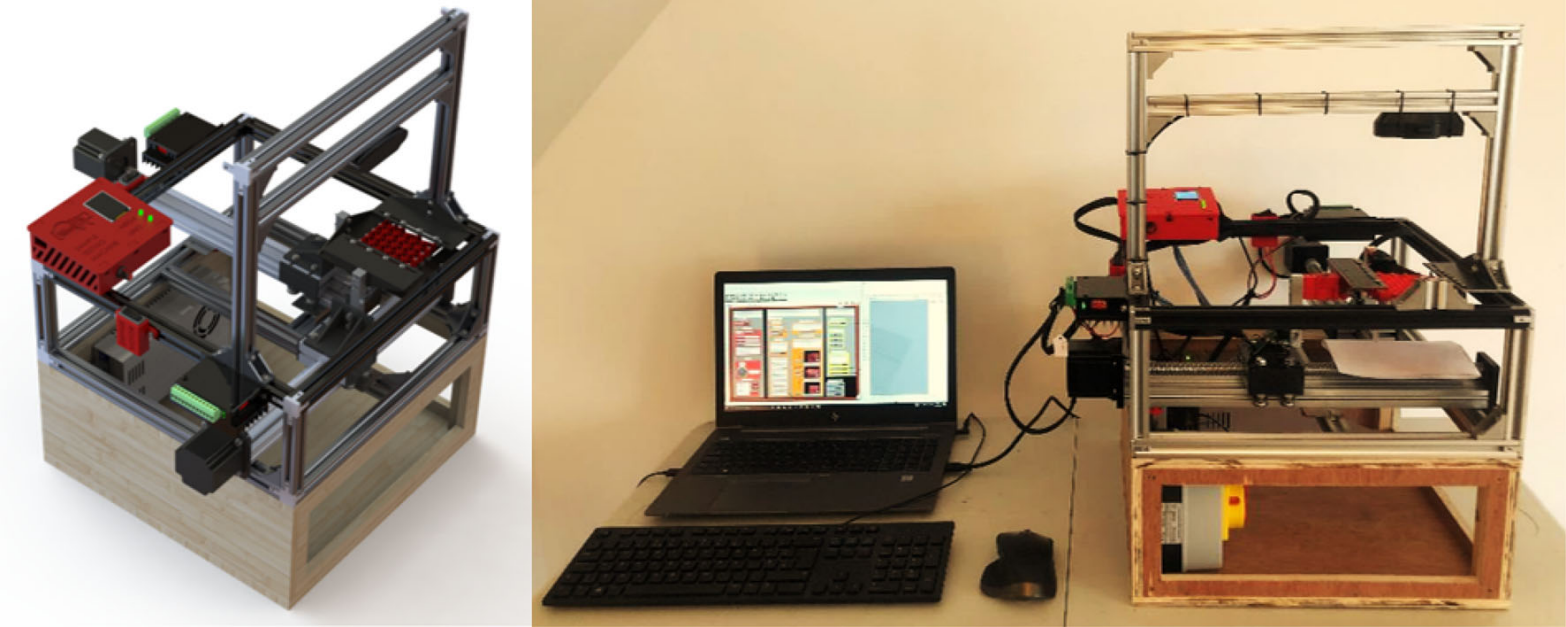
Rendering of test rig with completed physical setup with view of interface

The novel testing approach improves several key aspects of the methods explored in the previous sections (Fig. 4):

- *Combined tensile and compressive testing:* The testing rig permits both types of testing without the need for additional components or re-mounting of samples. This reduces time spent during test setup and improves consistency between tests.
- *Non-Orthogonal force application:* The new adjustable sample mount allows the force application angle to be altered which facilitates a range of tests to be carried out.
- *Automatic Data Management:* Grasshopper integrates with any computer to create folders for organising test files. Furthermore, test data is automatically organised into the assigned folder architecture

For the purposes of developing an efficient and effective testing workflow which can deliver real-time results, a control interface was also developed within the Rhino-Grasshopper definition, a full description of which can be found in other work developed by the authors [see 16].

## Summary of key results

**Fig. 5:**
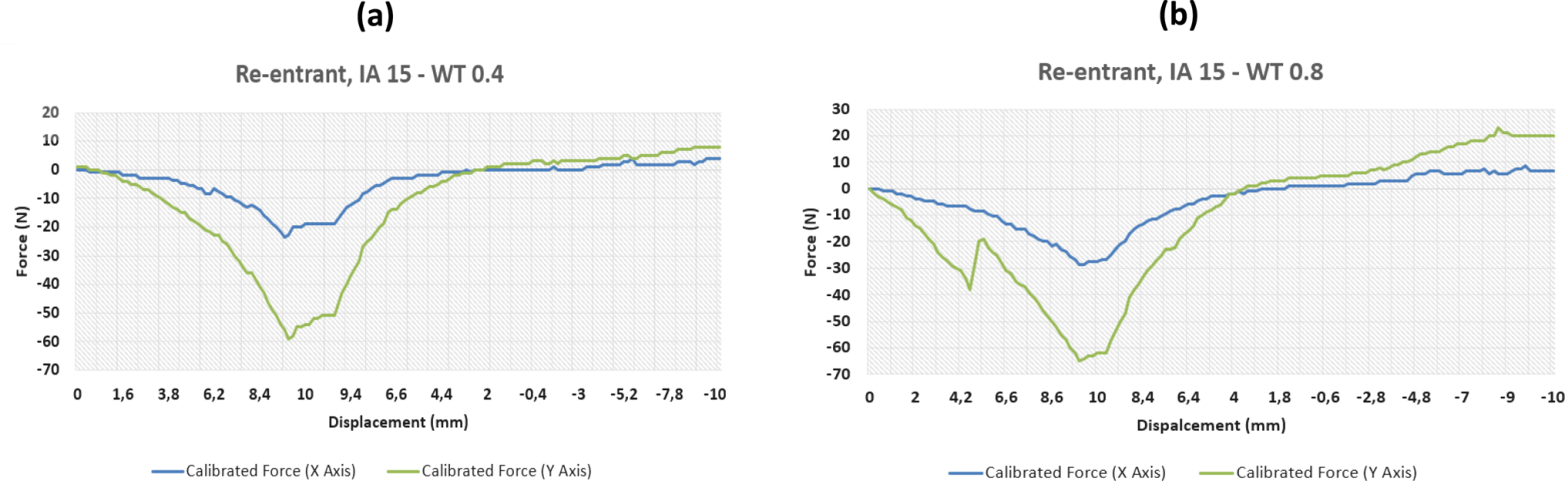
Results of force–displacement study for two re-entrant structure variants

**Fig. 6:**
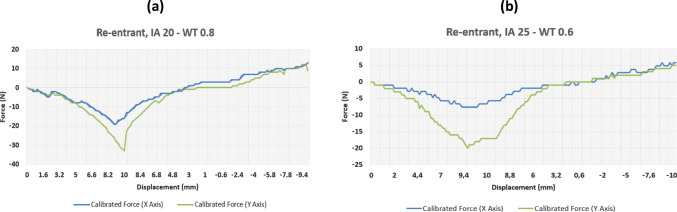
Results of force–displacement study for two re-entrant structure variants

**Fig. 7:**
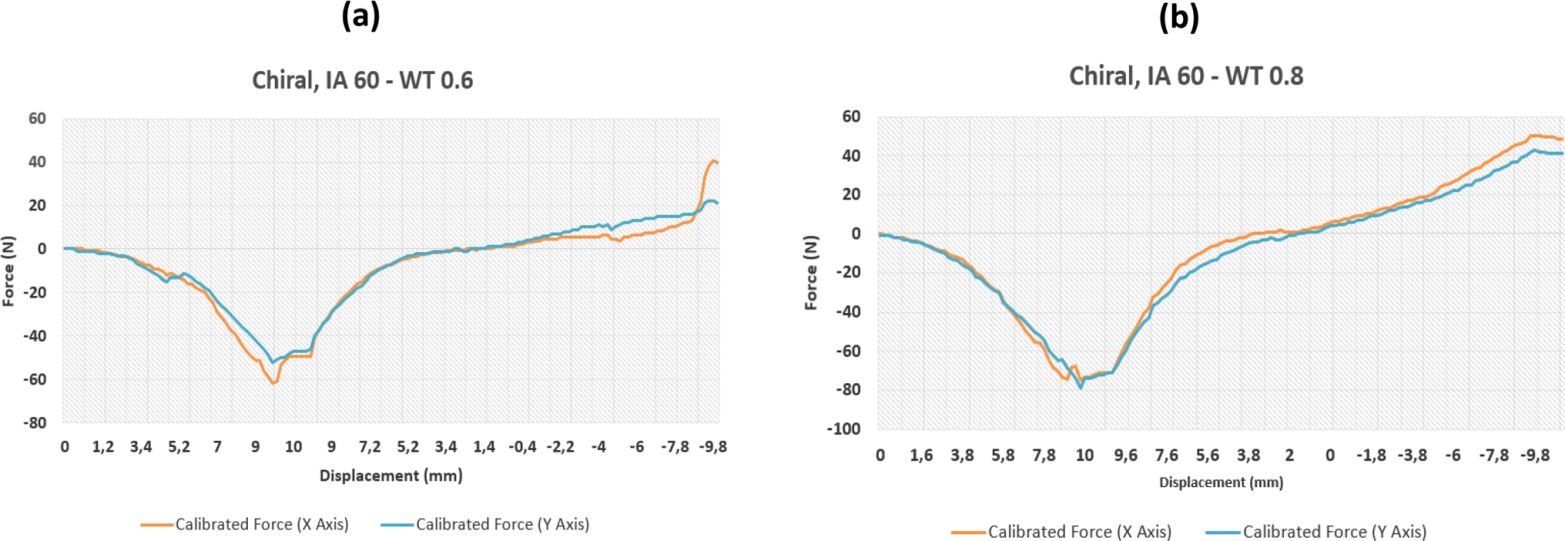
Results of force–displacement study for two chiral structures variants

**Fig. 8:**
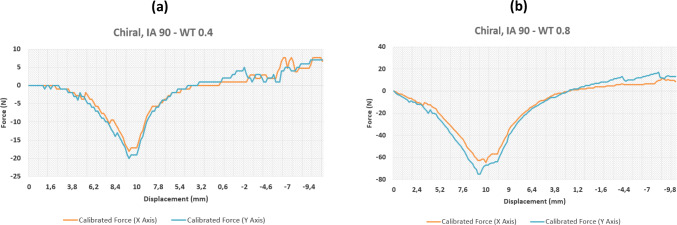
Results of force–displacement study for two chiral structures variants

**Fig. 9:**
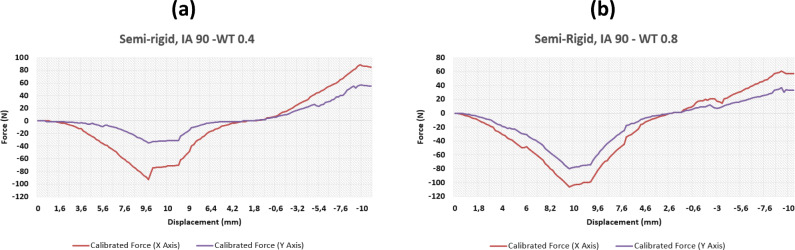
Results of force–displacement study for two semi-rigid structure variants

This section will focus on the key results from the testing of three conventional auxetics designs that were previously shown at Fig. 1; re-entrant, chiral, and semi-rigid. Various design parameters were edited in CAD including the interior angle (IA), and the wall thickness (WT) and then printed in TPU using a FlashForge 3D printer to create the separate samples. Each sample was 10 mm in depth and subjected to forces uniformly across all four sides. The results were analysed by means of load–displacement curves, which are shown in Figs. 5, 6, 7, 8 and 9. The graphs track a complete cycle from tension to compression whereby the left half of the graph (0–10 mm) shows the tension phase and the right half (10 to (− 10) mm) showing the compression phase.

### Re-entrant sample tests

The first tests were performed on re-entrant specimens. Starting with the lowest interior angle variable of 15°, several considerations can be drawn by comparing the results obtained with different wall thicknesses. For instance, the results obtained with the minimum and maximum wall thickness are reported in Fig. 5a and b, respectively, with a constant internal angle of 15°. Only small changes were recorded for both tension and compression forces at the two wall thickness extremes. This suggests that the largest overriding factor allowing the sample to sustain these loads may be the smaller interior angle, providing more mechanical freedom within the given deformation. There are some noted differences between the measured compressive forces, but this is only approximately 5–10 N across both axes.

More interesting conclusions can be drawn when changing the interior angle. Figure 6a and b shows the distinction between the recorded forces of the 0.8 mm wall thickness extreme for the re-entrant sample with a 20° interior angle as compared to 25° at 0.6 mm. Looking at the data for an interior angle of 25°, the lower recorded forces suggest even lower Young Modulus for that structure. Again, the lower wall thicknesses of this specimen led to slightly lower force readings as flexibility is enhanced when compared with other tests (not shown for brevity). On the other hand, the comparison between Figs. 5b and 6a shows the effect of changing the interior angle at fixed wall thickness. As can be noted, larger interior angles lead to lower Young’s modulus, confirming that this parameter has a higher impact on the structure stiffness with respect to the wall thickness.

### Chiral sample tests

The chiral results differed from the re-entrant results in one distinct way; they showed much greater uniformity in recorded mechanical behaviour across the *X* and *Y* axes, thus highlighting the specimen symmetry. Again, the interior angles and the wall thicknesses of the specimens were varied. Considering the results at an interior angle of 60°, at a fixed wall thickness of 0.8 mm, we can see a high level of uniformity in the recorded forces across both axes. As expected, thicker walls provide higher stiffness.

A similar pattern is also seen with an increase in recorded forces as the wall thickness is increased. Interestingly, the results for the chiral samples show a distinctly larger increase in measured forces across both axes than the re-entrant samples. Figure 5 shows an approximate increase by 20 N in tension and compression when different wall thicknesses are compared. On the other hand, when looking at a sample variant with a larger interior angle in Fig. 6b, the recorded forces are reduced markedly when compared to the 60° sample (Fig. 5b) suggesting that these samples have a higher degree of mechanical flexibility, echoing the re-entrant results. Interestingly however, doubling the wall thickness from 0.4 to 0.8 mm has a very large influence on the recorded forces (approximately three-fold), contrasting much of the re-entrant test data where the wall thickness has a marginal effect on the overall stiffness. Looking at Fig. 8a and b and comparing the graphs, a difference of about 60 N is recorded suggesting that this configuration is greatly affected by changes in wall profile.

Even for this chiral structure, the results with a different interior angle of 120° were analysed, even if not shown for brevity. The results were very similar to that of the 90° samples, being the structure stiffness more sensitive to the wall thickness than to the interior angle. Only small differences are recorded when the parts are in compression, where a marginally larger compressive force is recorded for the larger 0.8 mm wall thickness. The similarities between the 90° and the 120° results (not shown for brevity) suggest that the interior angle above a certain threshold does not make a huge impact of the amount of force sustained by the sample.

### Semi-rigid sample tests

The semi-rigid samples showed the same lack of uniformity as the re-entrant samples in terms of *X*–*Y* behaviour but showed much greater stiffness for both tension and compression when compared with most of the chiral and re-entrant samples. Considering the results shown in Fig. 9a and b, it is clear how much force the samples are sustaining as they are deformed. This is mostly ascribed to the increased rigidity of the samples due to their implicit geometric makeup, i.e., the samples are less skeletal and mostly solid compared to the chiral and re-entrant variants. While the chiral and re-entrant samples recoded forces at approximate maximums of 80 N, the semi-rigid samples sustained more than 100 N on the *x*-axis for both the wall thickness of 0.4 and 0.8 mm, predictably showing greater flexibility with the smaller wall thickness. The higher compression forces show that the sample is becoming more structurally uniform as the material is compressed together—a phenomenon described as “densification” [28]—becoming more uniform than the chiral and re-entrant samples.

These results constitute a starting point on which auxetics of different design can be systematically analysed. The novel setup that has been presented here provides an illustration of how a rigorous testing strategy can facilitate detailed comparisons of different kinds of componentry. In the next sections we will consider how such data may be integrated into design workflows to aid the development and generation of human-centred biomechanical products.

## Discussion: possibilities within novel design workflows

The key results discussed in the last section show clearly how small changes in the geometry of auxetic designs can sometimes greatly influence its mechanical behaviour when subjected to loading forces. But what are the implications of this in design terms? We showed in the previous sections how there has been a new focus on the applications of auxetics in a range of settings, particularly contexts that require a close consideration of human factors. This section will explore this in more detail by considering how the data acquired from mechanical analytics can inform key design decisions and may form part of novel design workflows developed for specific product specialisms.

**Fig. 10:**
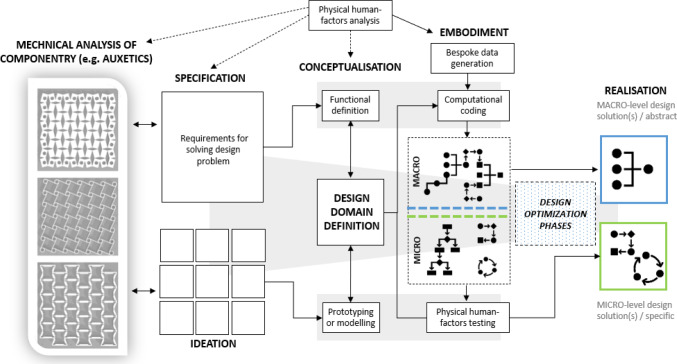
Design approach taken in the PRIME-VR2 research project illustrating how auxetics articulate with other forms of design data and inform design decisions

But how can such a treatment of componentry such as auxetics can factor into a human-centred design workflow? Here we can point to an illustrative example in the PRIME-VR2 research project (see https://prime-vr2.eu/) which is exploring the use of auxetic features as part of the creation of bespoke virtual reality controllers. Shown below in Fig. 10 is the methodological model that has been developed in which the status of mechanical analysis of componentry can be explored further and use as the foundation for exploring the differentials between the “macro” and “micro” levels of design output. This is the high-level version of the model, but it will be used as a starting point for establishing our synthesis between the technical outputs of computational design and the human-factors outputs which we have examined in the previous sections.

Narratively, the model follows the rough structure of a traditionally understood design methodology, starting with specification and ideation that articulates with a mechanical analysis of componentry and ending in a product realisation. Shown is how this interacts with a computational design workflow and creates design possibilities at both a micro level and macro level—discrete feature design against the larger structural architecture. The critical differences are seen as the solutions are being developed and the distinction between the macro solutions and the micro solutions which include a feedback loopthat integrates knowledge acquired from human-factors analysis.

1. **Mechanical analysis stage:** Exploration of mechanical properties of materials or componentry that may benefit design architecture.
2. **Specification/ideation stage:** The core requirements for the product are set out within the specifications which run in conjunction to initial ideation.
3. **Conceptualisation stage:** The pre-computational phase in which the functionality of the concepts is explored in the abstract and the design space constraints are more properly established.
4. **Embodiment stage:** Computation stage in which the abstract understanding of form and functionality explored in the first two stages become refined and are built into an algorithmic definition. At the stage, mechanical knowledge derived from testing becomes most important. The algorithmic definition has a macro stage optimising the larger structural form features and a micro stage generating the detailed form features and in-built functionality.
5. **Realisation stage:** Finalisation of design after computational design explorations. The design depending on the macro or micro strategy taken will be fully optimised around the specified constrains and any inputted data.

**Fig. 11:**
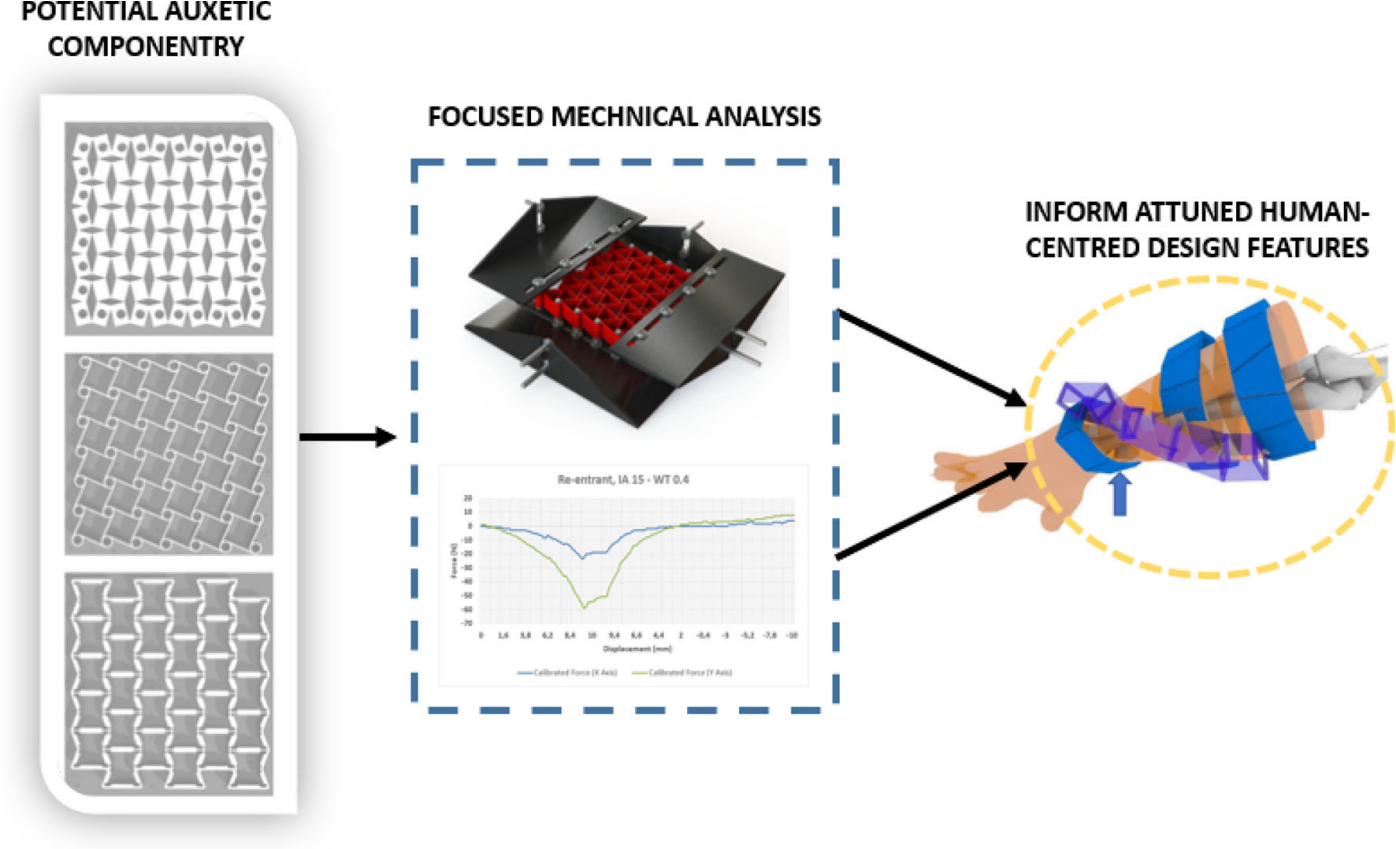
Illustrative example from PRIME-VR2 research project: conceptual framework for auxetics analysis informing design decisions in the creation of human-centred features

PRIME-VR2's research into bespoke therapy devices requires a human-centred approach with considerable focus on unique ergonomic requirements of users. Figure 11 illustrates how a macro level mechanical analysis can inform the design of componentry for specific features of the bespoke device e.g. a “spine” component acting as an anchoring point. Using a testing setup such as the one described in the previous sections, components that may be suitable for creating the component can be analysed with respect to likely motions and input forces, allowing for the optimization of the overall structure for the most effective mechanical performance. This kind of design workflow is particularly useful for the development of devices with specific functions and can also be integrated into computational approaches for the generation of optimised biomedical devices.

From what has been explored within this work and by other scholars, it is clear that auxetics have huge potential for applications within biomedical devices. Indeed, researchers such as Abby Paterson have taken this kind of analytics to new levels by creating tools in which bespoke features can be generated from specific human factors inputs articulating with mechanical performance preference settings. The mechanical analytics presented here are now being utilised in the development of bespoke biomedical devices and the workflow presented illustrates a vision in which features like auxetics may generate novel design solutions. While advanced testing setups are in some ways preferable due to their reliability of data output, this work demonstrates that good data can be generated from less technically advanced setups and reliable results can be gathered even using off-the-shelf componentry. This may aid designers in the early processes of creating devices that require conformity to complex mechanical motions or distinct geometries such as those of human anatomy of which auxetics may be useful.

## Conclusion

With a view to exploring human-centred design application of auxetics, this work presented the results from a novel setup to mechanically test auxetic components. Furthermore, our testing approached aims to unify a number of testing approaches into one umbrella system. Even if the presented setup was limited to “off-the-shelf” componentry, we sought to produce results similar to previous research efforts but provide novel solutions for the boundary condition question and unify particular methods such as optical analysis and force–displacement analysis. Firstly, we explored the emerging applications of auxetics in design and engineering with a particular focus on human-centred contexts where the materials were utilised to aid product functionality, usability or interaction with people. To establish this theme more solidly, we then described how auxetics are being utilised in a range of biomedical settings where conformity to human factors such as ergonomics is a vital concern. Secondly, the literature on auxetic testing approaches was examined in which it was determined that a range of mechanical testing strategies have been applied by researchers. These approaches were then loosely categorised as “conventional” and “advanced” setups and were subsequently discussed.

This was used as the foundation for the next phase of the work which focused on the novel testing strategy. We presented a brief summary of the development of the testing rig that was design explicitly to test small scale auxetic samples while carefully controlling the sample boundary as well as a specially configured interface created in Rhino-Grasshopper that provided full control of the testing procedures and data output. A range of tests were then conducted with this setup with a range of sample design variations which demonstrated the mechanical versatility of the different designs The data derived from the tests ultimately allowed for several conclusions to be drawn relating to the behaviour of auxetics of different build characteristics.

These results provided a foundation for the concluding discussions on how this kind of data can be incorporated into design development workflows. Particularly, we argue that the auxetics test data can be placed within workflows for developing human-centred products and present work from the PRIME-VR2 research project that illustrates a real-world application. With respect to the design of auxetics, a testing setup such as ours presents the opportunity for the quick analysis of additively manufactured componentry that is both useful and reliable, stimulating the creation of more elaborate auxetic structures.
